# Black Fungi and Hydrocarbons: An Environmental Survey for Alkylbenzene Assimilation

**DOI:** 10.3390/microorganisms9051008

**Published:** 2021-05-07

**Authors:** Noemi Carla Baron, Fernando Carlos Pagnocca, Ayumi Aquino Otsuka, Francesc Xavier Prenafeta-Boldú, Vânia Aparecida Vicente, Derlene Attili de Angelis

**Affiliations:** 1 Center for the Study of Social Insects, São Paulo State University (UNESP), Rio Claro 13506-900, SP, Brazil; noemicarlabaron@hotmail.com (N.C.B.); fernando.pagnocca@unesp.br (F.C.P.); ayumi.aquino@gmail.com (A.A.O.); 2 GIRO Program, Institute of Agrifood Research and Technology (IRTA), Torre Marimon, Caldes de Montbui, E08140 Barcelona, Spain; francesc.prenafeta@irta.cat; 3 Basic Pathology Department, Federal University of Paraná (UFPR), Curitiba 81531-980, PR, Brazil; vaniava63@gmail.com; 4 Division of Microbial Resources, CPQBA, University of Campinas (Unicamp), Campinas 13148-218, SP, Brazil

**Keywords:** toluene, biodegradation, melanized fungi

## Abstract

Environmental pollution with alkylbenzene hydrocarbons such as toluene is a recurring phenomenon. Their toxicity and harmful effect on people and the environment drive the search for sustainable removal techniques such as bioremediation, which is based on the microbial metabolism of xenobiotic compounds. Melanized fungi present extremophilic characteristics, which allow their survival in inhospitable habitats such as those contaminated with hydrocarbons. Screening methodologies for testing the microbial assimilation of volatile organic compounds (VOC) are scarce despite their importance for the bioremediation of hydrocarbon associated areas. In this study, 200 strains of melanized fungi were isolated from four different hydrocarbon-related environments by using selective methods, and their biodiversity was assessed by molecular and ecological analyses. Seventeen genera and 27 species from three main orders, namely Chaetothyriales, Cladosporiales, and Pleosporales, were identified. The ecological analysis showed a particular species distribution according to their original substrate. The isolated strains were also screened for their toluene assimilation potential using a simple and inexpensive methodology based on miniaturized incubations under controlled atmospheres. The biomass produced by the 200 strains with toluene as the sole carbon source was compared against positive and negative controls, with glucose and with only mineral medium, respectively. Nineteen strains were selected as the most promising for further investigation on the biodegradation of alkylbenzenes.

## 1. Introduction

Alkylbenzenes are a subset of aromatic hydrocarbons in which one or more hydrogen atoms from benzene have been replaced by alkyl groups of different sizes. The simplest member is toluene (C_6_H_5_CH_3_), in which a methyl group replaces a hydrogen atom from benzene. Toluene is a common bulk chemical used worldwide as a solvent for many substances such as paints, coatings, inks, adhesives, and cleaning agents [1] and as a gasoline additive for improving octane ratings. Toluene is also useful in the benzene production process and to obtain several polymers used to manufacture synthetic materials (e.g., nylon and polyurethanes), dyes, cosmetic and pharmaceutical products, and several specialized organic chemicals [2]. Toluene is relatively soluble and volatile, so it contributes to both water and air pollution. When inhaled, it acts as a central nervous system suppressor and may be lethal after exposure for one hour at 1800 to 2000 ppm *v/v* [3], and it may also cause chronic toxic effects at relatively low concentrations [4,5]. The American Conference of Governmental Industrial Hygienists (ACGIH) considers 20 ppm *v/v* as a threshold limit value (TLV) for toluene exposure to avoid occupational risks [6].

According to the International Tanker Owners Pollution Federation [7], the amount of oil spilled since 1970 has greatly decreased. However, the critical point mentioned by the ITOPF itself is that the statistics for small leakages (i.e., lower than 7 tons) are not precise due to the difficulty of obtaining reliable information on these events. In this context, most of the toluene and related alkylbenzenes are released to the ecosystems from gasoline and other oil products being spilled from storage tanks and pipelines [1,7]. Since these are mostly underground leaks, they remain undetected for long periods, severely affecting soil and groundwater and posing a significant ecotoxicological risk to all biological systems.

As a strategy to reduce atmospheric pollution, many countries supplement gasoline with ethanol [8]. In Brazil, commercial gasoline is a blend composed of 27% of anhydrous ethanol [9]. Toluene and other alkylbenzenes that constitute gasoline are miscible in primary alcohols such as methanol and ethanol, which are also soluble in water [10]. Thereby, the presence of ethanol allows the solubilization of high levels of alkylbenzenes [11], which, added to the aging and poor conservation of fuel storage tanks, results in leaks where ethanol carries gasoline alkylbenzenes to the soil aqueous phase. This enables alkylbenzenes to move through the soil matrix, increasing the probability of polluting underground water bodies and aquifers [4]. Such an environmental impact has been dubbed as BTEX pollution because of the predominance of benzene, toluene, ethylbenzene, and xylene isomers.

Bioremediation is an alternative technology to the usually more expensive and less environmentally sustainable physicochemical cleanup methods for treating contaminated areas. It is essentially based on the ability of microorganisms to metabolize recalcitrant and/or toxic compounds, such as aromatic hydrocarbons, by transforming them into substances with lower molecular weights that are more polar and, eventually, by completely degrading them into CO_2_ and H_2_O [12].

Filamentous fungi and yeasts have been widely studied for degrading harmful organic compounds [13,14,15,16]. Several studies point to the presence of melanized fungi (also known as black yeasts or black fungi) in environments rich in aromatic hydrocarbons, such as air biofilters for treating volatile hydrocarbons, soil contaminated with oil and gasoline spills, wood treated with creosote, and a coal-distilled fraction rich in phenolic compounds used historically as a wood preservative for railway ties and telephone poles [17,18,19,20,21]. The extremophilic nature of black fungi, in association with the recurrent isolations of their representatives in hydrocarbon-related environments, suggests their potential use in bioremediation processes.

Black fungi are a polyphyletic group that harbors several polyextremotolerant and oligotrophic species. The most evident adaptation of these ascomycetes is the production and accumulation of melanin in their cell walls. Melanin is a dark pigment that protects the cell and aids survival under a wide range of adverse conditions related to radiation and oxidative stress exposure [22,23]. Besides melanin, black fungi are able to biosynthesize other protective compounds such as mycosporines and mycosporine-like amino acids (MAAs) [22].

Gueidan et al. [24] suggested that ancestors of black fungi were originally oligotrophic organisms living on rock surfaces or subsurfaces. Currently, it is known that oligotrophic fungi can also grow in anthropogenic habitats such as glass, silicon, organic surfaces, metals [25], creosoted railway sleepers [26], and on phenolic compounds and aromatic hydrocarbons [21].

Various microbial enrichment assays based on a solid state-like protocol that used perlite as inert support incubated under a toluene atmosphere have consistently yielded melanized strains from the *Exophiala* and *Cladophialophora* genera, such as *E. xenobiotica*, *E. bergeri*, *C. immunda*, and *C. exuberans*, that are able to grow with toluene as the sole source of carbon and energy [18,20,27]. One of these strains, *Cladophialophora* sp. T1, later identified as *C. psammophila* [28], was successfully used in the biofiltration of toluene using inert packing materials [29]. As reviewed by Prenafeta-Boldú et al. [16], the assimilative toluene metabolic pathway in melanized fungi involves essentially the activity of cytochrome P-450 monooxygenase enzymes, which perform the oxidation of the methyl group as the first step in toluene degradation. Genomic studies such as that by Teixeira et al. [30] described how cytochrome P-450 genes are important for metabolizing aromatic compounds and in the process of adaptation to extreme environments. Blasi et al. [31] highlighted that cytochrome P-450 is one of the most overexpressed protein domains of *C. immunda* growing in the presence of toluene.

Considering the lack of effective and useful methods for screening microorganisms with the ability to degrade volatile organic compounds (VOC) such as BTEX and given the relevance of the topic to mitigate hydrocarbon pollution, this study aimed (1) to isolate and characterize the black fungal community of different hydrocarbon-related environments and (2) to test a reliable and simple method to screen and select fungi for their potential application in the bioremediation of pollution with alkylbenzene hydrocarbons. The proposed methodology is based on the analysis of biomass production by fungal isolates cultivated in a mineral liquid medium and incubated under toluene atmospheres inside desiccators.

## 2. Materials and Methods

### 2.1. Isolation and Preservation of Black Fungal Strains

Black fungi were isolated from four different environments naturally and artificially associated with hydrocarbons (Table 1): contaminated soil, plant material, water samples, and insects.

Three different enrichment and/or isolation techniques were applied on these four sample categories:

(a) Oil flotation technique: This isolation method is known to be highly selective for hydrophobic fungi such as black yeasts [32]. Soil samples were taken from a garage shop (city of Rio Claro, São Paulo, Brazil) contaminated with motor oil from vehicles and from land farming soil from an oil refinery (city of Paulínia, São Paulo, Brazil). This method was also applied on bark fragments of *Eucalyptus tereticornis* and exoskeletons of gynes and drones (winged females and males of leaf-cutting ants) of *Atta capiguara* and *Atta laevigata* obtained during the sexual reproduction period known as the “mating flight” (city of Botucatu, São Paulo, Brazil).
(b) Standard serial dilution method: Water samples from a river under the influence of an oil refinery (Atibaia River, São Paulo, Brazil) were processed according to the standard methods [33]. Fungi were isolated through serial dilutions of the samples, followed by plating on potato dextrose and Sabouraud agar (PDA and SA).
(c) Agar walk method: Living individuals of the previously mentioned winged females and males of leaf-cutting ants (*Atta capiguara* and *Atta laevigata*) were placed on malt yeast agar plates (MYA; peptic digest of animal tissue, 5.0 g; yeast extract, 3.0 g; malt extract, 3.0 g; dextrose, 10.0 g; agar, 20.0 g per liter of distilled water) added with cycloheximide (500.0 mg), chloramphenicol (200.0 mg), and streptomycin (200.0 mg), and were allowed to walk on the agar for one hour. Then they were removed, and the plates were incubated at 25 °C until the emergence of the black colonies.

Melanized fungal colonies were purified by subsequent transfers to new agar plates and preserved in slants containing 2% malt agar (MA; malt extract, 20.0 g; agar, 20.0 g per liter of distilled water) maintained at 4 °C and by the ultra-freezing technique at −80 °C. For this purpose, cultures were grown in 2.0 mL microtubes containing 500 µL of a 2% malt broth (malt extract, 20.0 g per liter of distilled water) for three to five days. Then, 500 µL of a sterile 20% glycerol solution and sterile glass beads (4 mm to 6 mm) were added to the microtubes and mixed manually. The tubes were placed into freezers at −20 °C overnight and then transferred to ultra-freezers at −80 °C.

### 2.2. Morphological Analysis

Filamentous black filamentous fungi were grown on 2% MA or PDA, while black yeasts were grown on SA. Incubations were performed at 25 °C for five to 10 days. The macroscopic analysis was carried out using a stereomicroscope, and the microscopic analysis was performed by the assembly of glass slides with cotton blue dye.

### 2.3. Molecular Identification

Genomic DNA was obtained following the modified extraction protocol by Möller et al. [34] using cetyltrimethylammonium bromide (CTAB). The ITS (Internal Transcribed Spacer) region of the ribosomal DNA was amplified using primers V9G and LS266 [35] or ITS1 and ITS4 [36] according to the following program: 94 °C for 3 min followed by 30 cycles at 94 °C for 30 s, 55 °C for 30 s, 72 °C for 1 min, and 10 °C forever. Amplicons were purified with the GFX PCR DNA kit and gel band purification (GE Healthcare, Chalfont Saint Giles, UK). The sequencing reaction was performed with primers ITS1 and ITS4 [36] according to the following program: 95 °C for 1 min followed by 28 cycles at 95 °C for 15 s, 50 °C for 45 s, 60 °C for 4 min, and 4 °C forever. The sequencing reaction products were purified with the DYEnamicTM ET Dye Terminator Kit (GE Healthcare^®^), following the manufacturer’s instructions, then loaded into an ABI3139 automated sequencer (Applied BiosystemsTM). The sequences were aligned and edited using software BioEdit version 7.0.5.3 [37] and then compared to other sequences from the Genbank (NCBI—National Center for Biotechnology Information) by using the BLAST (Basic Local Alignment Search Tool) [38] and from the CBS—Fungal Biodiversity Centre (The Netherlands). Phylogenetic studies were carried out using software Clustal W [39] for sequence alignment and MEGA X [40] for phylogenetic tree construction. The evolutionary history was inferred using the Maximum Likelihood method and the Kimura 2-parameter model [41].

### 2.4. Biomass Production in Toluene Atmospheres

**Figure 1:**
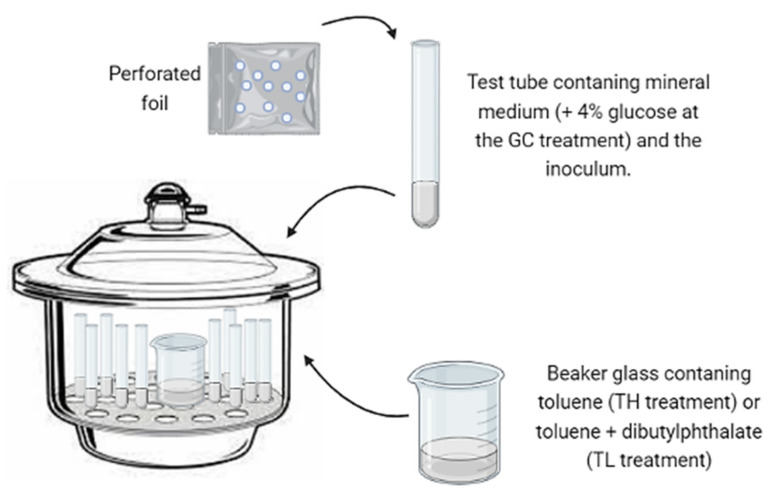
Schematic representation of the screening test to assess the potential of toluene assimilation by the melanized fungi. Test tubes containing fungal inoculum in a liquid medium were covered with perforated foil to allow gas exchange inside the desiccators. A beaker glass containing pure toluene or toluene diluted in dibutyl phthalate (DBP) were placed inside the desiccators in order to supply toluene as the sole source of carbon and energy via the gas phase at high and low concentrations, respectively.

The isolated strains of black fungi were grown on 2% MA for seven days. Test tubes (10 mL) previously weighted on an analytical balance were filled with 3 mL of a mineral medium composed of a macronutrient solution (KH_2_PO_4_, 4.5 g; K_2_HPO_4_, 0.5 g; NH_4_Cl, 2.0 g; MgSO_4_·7H_2_O, 0.1 g; distilled water, 1000 mL) combined with a micronutrient solution (FeCl_3_, 120 mg; H_3_BO, 350 mg; CuSO_4_·5H_2_O, 10 mg; KI 10 mg; MnSO_4_·H_2_O, 45 mg; Na_2_MoO_4_·2H_2_O, 20 mg; ZnSO_4_·7H_2_O, 75 mg; CoCl_2_·6H_2_O, 50 mg; AlK(SO_4_)·12H_2_O, 20 mg; CaCl_2_·2H_2_O, 13.25 g; NaCl, 10.0 g; distilled water, 1000 mL) in a proportion of 1000 mL: 2 mL, respectively. The tubes were inoculated with a small amount of mycelia using an inoculation needle. The inoculum mass was undetectable by an analytical balance, and the mycelia were carefully collected to avoid contact with the agar culture medium. After the inoculation, the tubes were covered with perforated foil to allow gas exchange and incubated inside three desiccators (12 L of air volume each) under the following conditions: (1) positive growth control (GC), a mineral medium with 4% glucose as the carbon source; (2) toluene atmosphere at a high concentration (TH), a mineral medium and an atmosphere with toluene as the sole carbon source, provided by an uncovered flask containing 15 mL of pure toluene; (3) toluene atmosphere at low concentration (TL), a mineral medium and a toluene atmosphere created by placing a flask containing 15 mL of toluene and 15 mL of dibutyl phthalate (DBP); (4) negative control (NC), only a mineral medium to monitor the endogenous growth (Figure 1). According to the volatility, and the water/air and water/DBP partition coefficients of toluene at normal conditions [42], the initial estimated concentration of this compound at the TH and TL incubation conditions was 37,000 and 140 ppm *v*/*v*, respectively.

The desiccators were closed and incubated at room temperature for 25 days. After the incubation, the biomass growth was assessed by measuring the dry weight. Test tubes were dried at 105 °C for 24 h then weighed on an analytical balance. The biomass values were obtained by calculating the difference between the final and initial weights of the tubes.

### 2.5. Selection of Strains with Toluene Assimilation Potential

For any given fungal strain, the harvested biomasses in terms of dry matter from the previously described incubations (GC, TH, LH, and NC) were used to calculate the growth ratios so to identify the strains that displayed the best growth potentials using toluene as the sole carbon source:

GC/NC ratio: amount of biomass produced on glucose (GC) divided by the biomass obtained with the negative control (NC). This ratio reflects how much more biomass is grown on a readily-used substrate versus the endogenous growth with no carbon source and indicates that a given strain is able to grow on a readily biodegradable substrate under the tested laboratory conditions.

TH/NC ratio: amount of biomass produced in the toluene atmosphere (TH) divided by the biomass obtained with the negative control (NC). The higher this value is, the greater the likeliness of a given strain assimilating toluene at high concentrations.

TL/NC ratio: biomass production in the presence of toluene and dibutyl phthalate (TL) divided by the biomass from the negative control (NC). The higher this value is, the greater the likeliness of a given strain using toluene at low concentrations. Comparisons between TH/NC and TL/NC might indicate toluene toxicity.

Taking into account the ratios of biomass produced when supplying a carbon source (GC, TL, TH) in relation to the negative control (NC), the strains were ranked in four categories: category 1, when the growth with a carbon source relative to the negative control (TG/NC, TH/NC, and TL/NC) is below 2; category 2, when the TG/NC, TH/NC, and TL/NC ratios range from 2 to 5; category 3, when the TG/NC, TH/NC, and TL/NC ratios range from 5 to 10; category 4, when the TG/NC, TH/NC, and TL/NC ratios are greater than 10. Strains displaying TH/NC and TL/NC ratios in categories 3 and 4 were considered potential candidates for assimilative toluene biodegradation.

### 2.6. Multivariate Ecological Analysis

The multivariate analysis of the occurrence of fungal ribotype sequences was performed by means of the web-based biostatistics package Visualization and Analysis of Microbial Populations Structure (VAMPS) using the UNITE fungal genome database [43]. This software was also used for calculating the fungal species richness and biodiversity indices of the samples. A principal coordinate analysis (PCoA) analysis using the Bray–Curtis distance metric was performed on the sequence data obtained from the isolated fungi, and the results were visualized in a 2D plot using the VAMPS package.

## 3. Results and Discussion

### 3.1. Isolation and Identification

A total of 200 strains were obtained from the isolation program carried out in hydrocarbon-related environments: contaminated soil, plant material, water samples, and insects. These strains were identified on morphological grounds, and identifications were confirmed through molecular means for 138 of them (Table S1). A total of 17 genera and 27 species were recognized by morphological and molecular analyses, belonging to the orders Chaetothyriales (86 strains), Pleosporales (52 strains), Cladosporiales (47), Capnodiales (5 strains), Microascales (1 strain), Xylariales (1 strain), and Venturiales (1 strain). The remaining seven strains were grouped as “melanized filamentous fungi” due to the lack of molecular data and distinctive phenotypical characteristics for an accurate identification.

**Figure 2:**
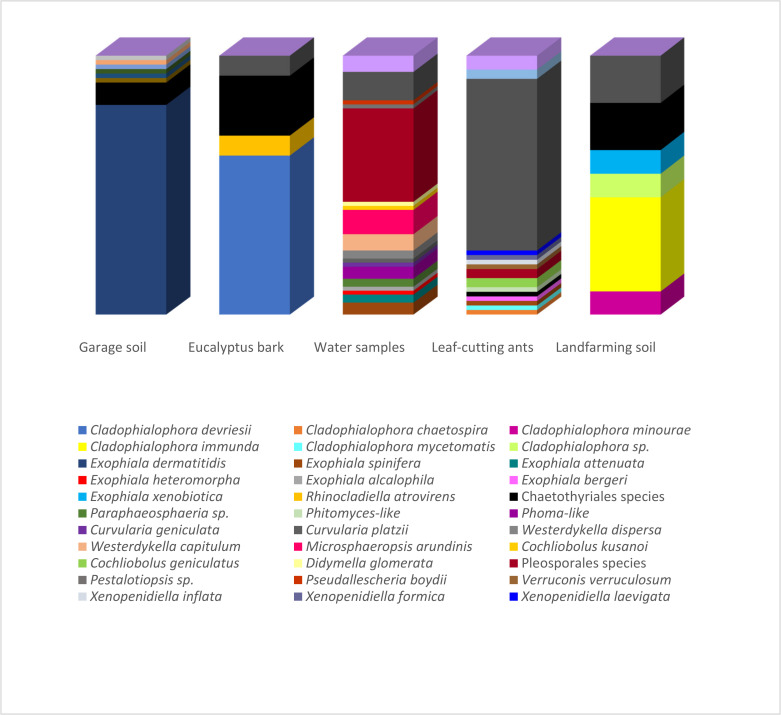
Relative abundance of strains obtained from the different hydrocarbon-related environments. Strains isolated from the garage soil, *Eucalyptus* bark, landfarming soil and leaf-cutting ants were obtained using the oil flotation technique (Satow et al., 2008). The ants were also submitted to the agar walk method. Strains recovered from the water samples were obtained by standard serial dilution according to Clesceri et al. (1998).

Figure 2 presents the correspondence between the isolated species and their environments. Species richness estimators and biodiversity indices on the isolated fungi are summarized in Table 2. The highest number of isolates (over 40 strains) was obtained from samples of the hydrocarbon-associated soil and water using the oil flotation and the pour-plate methods, respectively. However, while all identified strains from the garage soil were associated with *Exophiala dermatitidis* (Chaetothyriales), the highest observed richness (25 species) and biodiversity indices were observed in the water samples under the influence of an oil refinery. The strains identified from land farming and bark samples were also primarily related to the Chaetothyriales, which comprised the highest number of strains identified in the present study.

Significant advances in the ecophysiology of Chaetothyrialean fungi have been achieved in the last decades [30]. This clade encompasses species that have been isolated recurrently from both clinical patients and hydrocarbon-rich environments. The connection between these two apparently distinct and highly-specific ecological traits is still a matter of discussion, and isolation campaigns such as that of the present study might provide deeper insights into the evolution of this group [44,45]. A genomic study by Moreno et al. [46] suggested an overlap between metabolic pathways used for nutrient acquisition in extreme environments and pathogenicity factors. Furthermore, it was found that black yeasts are able to undergo contractions and expansions of their genomes to increase survival according to changing environmental conditions [30,47]. Ant domatia-associated fungal species present smaller genomes than other related species in the Chaetothyriales, which might occur due to the specialized symbiosis with the insects [47]. Three genera belonging to the Herpotrichiellaceae family (Chaetothyriales) were identified among the isolates: *Cladophialophora, Rhinocladiella*, and *Exophiala*. A phylogenetic analysis carried out with sequences of the representative species of these three genera is shown in Figure S1.

*Cladophialophora* is a monophyletic genus that comprises the *bantiana* and *carrionii* clades [30,48]. Their representatives are common human pathogens, but non-virulent environmental strains have also been reported, such as *C. yegresii* [49], *C. hostae*, *C. proteae*, and *C. scillae* [50], *C. pseudocarrionii* [51], *C. lanosa* [52], and *C. psammophila* [28]. In this study, the species *C. devriesii*, *C. mycetomatis*, *C. chaetospira*, *C. minourae*, and *C. immunda* were identified. Although commonly described as a human pathogenic black yeast, strains of *C. devriesii* have also been isolated from environmental samples [48], as found in this study with the eight strains from this species obtained from *E. tereticornis* bark. It is known that this evergreen tree is rich in natural resins that contain volatile phenolic compounds and hydrocarbons [53,54]. It is important to highlight that this is the first report of *C. devriesii* from *Eucalyptus*.

*Cladophialophora mycetomatis*, also found in this study, is an uncommon species originally described from the human foot after trauma with the *Opuntia* spine (Cactaceae) in Mexico. To date, only two publications reported its isolation: Badali et al. [48], who described the species working with strains CBS 122,637 (clinical) and CBS 454.82 (environmental), and Nascimento et al. [27], who described the isolation of this species from babassu coconut shells. In this study, one strain of *C. mycetomatis* was obtained from the body of an *A. laevigata* drone. This is the first report of this black yeast in insects and, in particular, in leaf-cutting ants of the Attini tribe. Despite the first report from a human mycetoma, only environmental strains have been obtained since then, with no disease association.

Napolitano and Juaréz [55] were the first to suggest the use of hydrocarbons from the cuticle of insects as the sole carbon source for the growth of entomopathogenic fungi on *Triatoma infestans*. Regarding the black fungi, several authors concluded the existence of a straight relationship between their presence and the availability of hydrocarbons as a carbon source [17,18,20]. Attili-Angelis et al. [56] described two new species of *Phialophora* associated with *Atta* spp. and addressed questions on the diversity of ant-associated Chaetothyriales and their ecological aspects. A correlation of the hydrocarbons present in the insect cuticle with the occurrence of these fungi on the ant exoskeleton was suggested, thus reaffirming the hypothesis that these molecules are a potential key for survival in this environment. In the present study, one strain of *C. chaetospira*, originally described as a saprophytic species [48], was isolated from ants (Figure 2).

Landfarming soil is an environment rich in petroleum hydrocarbons that receives specific management aiming at the removal of these pollutants. From these samples, one strain of *C. minoure* and four of *C. immunda* were isolated. The first is a saprophytic fungus occurring on plant debris [48] and not commonly associated with hydrocarbon-contaminated environments, unlike *C. immunda*, as its own name suggests an association with pollution [48]. In fact, toluene assimilation in this later fungus has been studied at the transcriptomic level [31].

A total of 57 strains were identified as *Exophiala*, with the representative species being *E. dermatitidis* (47), *E. spinifera* (4), *E. attenuata* (2), *E. bergeri* (1), *E. heteromorpha* (1), *E. alcalophila* (1), and *E. xenobiotica* (1). All the *E. dermatitidis* strains were isolated from the garage soil (Figure 2), indicating a high selectivity of the substrate and adaptation of the species to petroleum hydrocarbons. This species is related to human infections, but Sudhadham et al. [57] pointed out that birds, frugivorous bat feces, and fruits in tropical environments might be its environmental origin. The authors suggested that the entry into the human habitat came from the ingestion of wild fruits carrying the propagules of the species.

*Exophiala alcalophila*, *E. heteromorpha*, *E. attenuata*, and three of the four *E. spinifera* strains were obtained from the water samples (Figure 2), indicating a higher diversity of the genus in this environment. The last strain of *E. spinifera* and the single strain of *E. bergeri* were isolated from the leaf-cutting ants, while *E. xenobiotica* was recovered from the landfarming soil (Figure 2). The *Exophiala* genus is divided into several clades [58]. *Exophiala heteromorpha* is grouped in the *dermatitidis* clade, while *E. xenobiotica* and *E. bergeri* belong to the *spinifera* clade. These two clades contain the main lineages recognized as human pathogens, except for *E. xenobiotica*, originally isolated from sites contaminated with aromatic hydrocarbons [17]. No records involving *E. attenuata*, a representative of the *mesophila* clade, and *E. alcalophila*, which belongs to the *alcalophila* clade, with hydrocarbon-associated environments were found in the literature.

Two strains were identified as belonging to the *Rhinocladiella* genus, one of *R. atrovirens* and the other of *R. similis*, which are closely-related species. The *Rhinocladiella* genus is a synanamorph of *Exophiala*, i.e., both anamorphic species can occur at the same teleomorph. Del Palacio-Hernanz et al. [59] described the pathogenic nature of *R. atrovirens*; however, recent studies have been published where the species is placed as a saprotroph in the decomposition of pine wood [60,61].

The extremotolerance of melanized fungi is unquestionable. Ruibal et al. [62] isolated melanized fungi, including representatives of the Chaetothyriales order, from rock surfaces, where extreme adverse conditions such as high exposure to solar radiation and temperature, low nutrient availability, high electrolyte concentration, and low relative humidity are common. Besides the inhospitable rock surfaces, hydrocarbon-related environments such as sites contaminated with oil and its byproducts and creosote-treated wood are a great source for the isolation of these fungi [32,63], but they may also be found in unpolluted sites such as soil and vegetal debris [64]. Therefore, the general idea of their pathogenic nature must be revised, and biohazards must be assessed carefully for every new Chaetothyrialean species that displays a biotechnological potential.

The recurrent isolation of these fungi from environmental samples naturally or artificially exposed to hydrocarbons and related substrates corroborates the hypothesis of ecological dualities. This argues that strains isolated from the environment but identified as pathogenic and/or opportunistic due to a high genetic similarity might have an ecological role not related to virulence. That is, ecological niches between clinical and environmental strains related to hydrocarbons would be clearly differentiated, and the later strains would not cause infections [28].

The order Pleosporales presented the second largest number of representatives (52), isolated from all substrates (Figure 2). Pleosporales encompass a quarter of the Dothideomycetes class representatives, being the largest of its orders [65]. Pleosporalean species may occur in several habitats as epiphytes, endophytes, parasites of leaves and stems, or hyperparasites of fungi or insects, and in association with lichens or saprobes [66]. Reports of opportunistic strains are rare, which favors the exploration of their biotechnological potential, including their xenobiotic degradation capacity, which is still poorly studied in this group.

Most of the Pleosporalean strains identified in this study (44 strains out of 52) were isolated from water samples from the river under the influence of an oil refinery (Figure 2), and the eight remaining strains were obtained from the exoskeleton of leaf-cutting ants. Fungal metabolic diversity is quite significant, and the presence of alternative carbon sources that might also be toxic, such as the hydrocarbons present in these environments, could act as triggers to activate biodegradation pathways linked to detoxification and even assimilatory metabolism.

The Pleosporales anamorphs are mostly coelomycetes, but they may also be hyphomycetes. *Phoma* and its relatives are the most common anamorphs [66]. Boerema et al. [67] proposed a morphology-based classification of *Phoma* into nine sections: *Phoma*, *Heterospora*, *Macrospora*, *Paraphoma*, *Peyronellaea*, *Phyllostictoides*, *Pilosa*, *Plenodomus*, and *Sclerophomella*, which are precisely described in the “*Phoma* Identification Manual” [68]. The manual contains 223 descriptions of specific and infra-specific taxa and over 1000 synonyms in other coelomycetes genera [69]. Currently, the phylogenetic analysis of *Phoma* and its relatives shows the existence of several families, indicating the polyphyly of several genera and making the identification of representatives of this group more consistent [66]. In this study, 27 Pleosporalean strains were identified belonging to the genera *Westerdykella* (6), *Microsphaeropsis* (6) *Cochliobolus* (3), *Curvularia* (2), *Paraphaeosphaeria* (2), *Epicoccum* (1), *Didymella* (3), *Pithomyces*-like (1), and *Phoma*-like (3) (Figure 2; Figure S2). The 25 remaining strains were characterized as Pleosporales representatives by their morphologies. The absence of morphological characteristics (sterile colonies) and the low resolution of the DNA sequences hampered the genus/species-level identification.

Six strains were identified as *Westerdykella* (Sporormiaceae)*,* including *W. capitulum* (4) and *W. dispersa* (2). The genus includes ubiquitous species occurring in manure as endophytes or in soil as saprophytes [70]. *Cochliobolus kusanoi* (1) and *C. geniculatus* (2) were isolated from water samples and the bodies of an *A. capiguara* drone and an *A. laevigata* gyne, respectively. Two *Curvularia* strains, namely *C. platzii* and *C. geniculata*, were also obtained from the water samples. The presence of *Cochliobolus* and its anamorphs *Curvularia* and *Bipolaris* had already been reported in Attini ants [71]. The three genera are members of the Pleosporaceae family and form a complex with well-known phytopathogenic species, especially of the Poaceae family (grass). Also, the *Pithomyces* genus is found in this family. One strain was morphologically identified as *Pithomyces*-like, a cosmopolitan genus found in soil and debris [72].

Six strains recovered from the water samples were identified as *Microsphaeropsis arundinis*, a member of the Montagnulaceae family. Alves et al. [73] described this species as an endophyte and ubiquitous. Costa et al. [74] reported its common presence in the mycobiota of Brazilian mangrove plants on the northeastern coast. Luo et al. [75] observed the production of specific sesquiterpenes by this species, referred to as “arundinols”. From the same family, two strains of *Paraphaeosphaeria* sp. were isolated from the same substrate.

*Didymella glomerata* was the representative of the Didymellaceae family, having been isolated from the water samples in this study. As with most of the Pleosporales, this species has been obtained for a long time from senescent leaves of deciduous trees [76,77]. Three other strains from the same substrate were morphologically identified as *Phoma*-like due to the presence of pigmented conidia, unlike what occurs with *Phoma* representatives. Molecular data from the ribosomal DNA did not clearly define the specific identification of the strains.

A total of 52 isolates were identified as representatives of the orders Cladosporiales (47) and Capnodiales (5). The Cladosporiales order was recently proposed, as well as the family Cladosporiaceae, in which it is included [78]. Members of Cladosporiales were previously treated as Capnodiales. Both orders belong to the Dothideomycetidae subclass and comprises epiphytes, endophytes, and saprophytes species, occurring in association with algae (forming lichens) or as parasites of fungi and animals [78,79]. In this study, all the Cladosporiales isolates belong to the *Cladosporium* genus. The identification to specific level was not possible since the ITS sequencing was not enough to define all species. This is a very complex and heterogeneous genus comprising hyphomycetes with a typical recognizable coronate scar type. The morphological differentiation among species is often confusing, so molecular tools are of great importance. However, even using DNA sequencing, the comparison of sequences from only one genic region may not be reliable enough for precise identification. Data from a phylogenetic analysis with some strains show that the species are allocated at the *cladosporioides* complex [80] within the *Cladosporium* genus (Figure S2).

Among the 47 *Cladosporium* strains, 37 were obtained from the exoskeleton of leaf-cutting ants. Rodrigues et al. [81,82] previously reported the presence of this genus on ants. Pagnocca et al. [83] also found the predominance of *Cladosporium* strains from *Atta* gynes, pointing out that these insects could act on its dispersion, but no interactions were shown between this genus and the symbiont fungus cultivated by the ants.

The other 10 *Cladosporium* strains were isolated from the water samples under the influence of an oil refinery (7), from the landfarming soil (2), and one strain from *E. tereticornis* bark (Figure 2). *Cladosporium* conidia are the most common fungal component isolated from the air [84]. It is a cosmopolitan genus with species found in all kinds of plants, debris, soil, food, paints, textiles, and any other organic matter [85]. Considering the substrates studied herein, its occurrence was quite expected. The highest number of *Cladosporium* species are clustered in the *cladosporioides* complex, and several studies report their common isolation from different environments [86,87,88], explaining the prevalence of species from this complex in the studied samples.

Representatives of the *Xenopenidiella* genus were obtained as members of the Capnodiales order. Only three strains were recovered from the cuticle of *A. laevigata* leaf-cutting ants (Figure 2). *Xenopenidiella formica* and *X. inflata* were isolated from drones, while *X. laevigata* was isolated from a gyne. The genus, very similar to *Penidiella,* was described by Quaedvlieg et al. [89] as a saprophytic on leaf litter. The presence of *Xenopenidiella* species associated with the ants is possibly related to their habit of foraging fresh plant matter into the nest. The molecular analysis carried in this study revealed that these three strains could represent novelties to science. Their description was made by Duarte et al. [90].

Besides these orders, one strain of *Pseudallescheria boydii* (from water samples—Microascales), one of *Pestalotiopsis* sp. (from water samples—Xylariales), and one of *Verruconis verruculosum* (drone of *A. laevigata*—Venturiales) were also isolated. The Microascales order includes saprophytic fungi commonly found in soil [91]. *Pseudallescheria boydii* is originally a saprophyte often isolated from agricultural soils and contaminated water; however, it has been reported from clinical samples, especially in immunosuppressed patients [92]. It is also found in hydrocarbon-related environments such as diesel pipelines and soil with oil residues, being able to degrade oil and its byproducts besides biodiesel [93,94,95,96]. Xylariales is a monophyletic order with more than 92 genera [97]. The *Pestalotiopsis* genus is widely spread in tropical and temperate regions and is an important plant pathogen. More than 235 species are described in this genus, and they are named according to the hosts they are associated with [98,99]. Venturiales and the Sympoventuriaceae family were described by Zhang et al. [66] in order to accommodate members of the Venturiaceae family traditionally grouped with Pleosporales. *Verruconis* and *Ochroconis* are related genera that belong to the Sympoventuriaceae family. *Verruconis* spp. are thermophilic, while *Ochroconis* spp. are mesophylic. Both genera contain clinical and environmental strains reported in the literature [100], corroborating the ecological duality already mentioned for melanized fungi.

### 3.2. Fungal Community Structure

**Figure 3:**
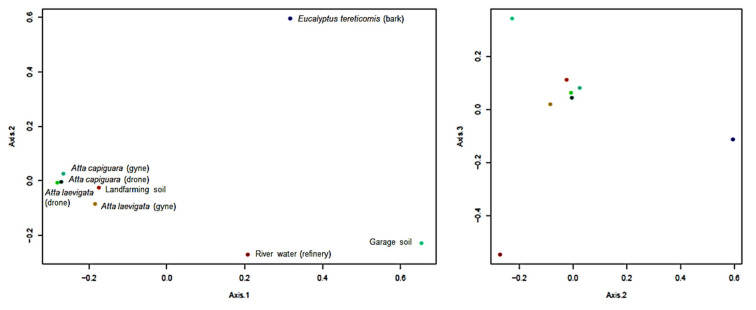
Two-dimensional (2D) plots of a principal coordinate analysis (PCoA, Bray–Curtis distance) on the relative abundance of melanized strains isolated from different hydrocarbon-associated environments, based on the analysis of their ITS rDNA sequences (Encompassed variance: 32.86% in Axis 1, 20.40% in Axis 2, and 17.72% in Axis 3).

The similarities between the community structure of the isolated black fungi in the studied samples were analyzed by multivariate PCoA. The total variance explained by the three first axes amounted to 70.983%, and sample scores were grouped around the main cluster formed by those fungal communities obtained from the cuticle of ants (Figure 3). Such relatively similar fungal communities from ants were observed even though were isolated from different individuals and species (drones and gynes of *A. capiguara* and *A. laevigata*) using two very distinct isolation methods (oil flotation and agar walk). All insect samples had in common the predominance of *Cladosporium* spp., but some other isolates were shared among at least two individuals (*Xenopenidiella* sp. and Pleosporales sp.) or were single isolations (*Exophiala* and *Cladophialophora* spp., and *Verruconis* sp.). The cuticle of these ants is a highly hydrophobic environment that also contains volatile pheromones used for communication by these social insects. These signaling molecules include aromatic compounds (e.g., pyrrole and pyrazine) and long-chain hydrocarbons (alkanes and alkenes) [101]. The black fungi isolated from the landfarming soil with a history of oil pollution yielded a very limited number of strains, some of which had previously been recognized as hydrocarbon degraders (e.g., *C. immunda* and *E. xenobiotica*; Figure 2; Table S1).

Conversely, the community structure of melanized fungi from the garage soil, river waters, and bark were clearly distinct from each other and in relation to those from insects. Strains from the garage soil were obtained by the oil flotation technique, and practically all of them were identified as *E. dermatitidis*. Apparently, the profile of oil-derived hydrocarbons in this environment exerted a very selective pressure on fungal biodiversity, favoring this particular taxon. A possible reason for this is the accumulation of motor oil, lubrication additives, and toxic metals that might restrict microbial growth to tolerant species. It could also be that the selectivity of the oil flotation technique resulted in a positive bias towards the isolation of this particular species; however, the oil flotation was also used in other samples (landfarming soil, bark, and ants) and gave very different results in terms of biodiversity and identity of predominant species. Similar to the garage soil, fungi from *Eucalyptus* bark were strongly dominated by a single species, in this case, *C. devriesii* (Figure 3). The bark of *E. tericornis* is highly hydrophobic and contains several hydrocarbons, which are mainly present as resins [53,54].

Interestingly, the water samples related to the refinery were characterized by the highest melanized fungal biodiversity. This is to be expected when using the less-specific pour-plate methods, but the isolation from water samples of a large number of melanized fungi, which in principle grow relatively slowly on culture media and have generally been associated with solid surfaces (rocks, bark, cuticle, etc.), is quite puzzling. Thus, it is worth examining whether or not melanized fungi could be used as bioindicators of present or past pollution events with petroleum hydrocarbons in water bodies.

### 3.3. Growth Specificity Experiments

This test aimed to select fungi able to produce biomass in a selective environment containing toluene as the sole source of carbon and energy at high and low concentrations. The biomass yield values and ratios obtained with the different growth conditions tested are compiled in Table S1, but a subset with the most promising strains in terms of growth in the presence of toluene has been summarized in Table 3. The analysis showed that growth on glucose usually yielded more biomass than on toluene because the former is a readily available sugar (the GC/NC ratio is generally greater than TL/NC or TH/NC; Table 2). In most cases, when there was significant growth on toluene, the TL/NC ratio was the same or higher than the TH/NC ratio, which points to the toxicity of this compound at air saturation for certain isolates. Although toluene is responsible for extensive cellular damage, mainly at the membrane, the data showed that several of the isolated strains were able to tolerate and even grow on toluene at air-saturation conditions.

Concerning the candidate strains for toluene assimilation (Table 3), *W. dispersa* A55, *Pestalotiopsis* sp. A234, and *Cladosporium* sp. N34 deserve special attention due to their high biomass production ratio relative to the endogenous control, with both high and low toluene concentrations (TH/NC and TL/NC). The strains *C. devriesii* D4, Chaetothyriales sp. D15, and Pleosporales sp. NR7 were only able to increase more than tenfold the biomass production compared to NC at high toluene concentrations (TH). On the other hand, the Chaetothyrialean strains *C devriesii* D5 and *E. dermatitidis* D180 exhibited the same pattern but only at low toluene concentrations (TL). All the other *Cladosporium* sp. strains (N21, N85, N92, N101, and F18), *E. dermatitidis* D153, and the melanized filamentous fungus F21 displayed moderate TL/NC and TH/NC ratios and are also promising strains for toluene assimilation. Finally, the TH/NC ratio for the strains *E. dermatitidis* D31 and Pleosporales sp. A393 was close to 10, and the melanized filamentous fungus A304 showed a similar condition but at low toluene concentrations (TL/NC).

Interestingly, three orders are predominant among the 19 selected strains: 5 representatives of Pleosporales, 6 Cladosporiales (all *Cladosporium* spp.), 6 Chaetothyriales, and 2 unidentified melanized filamentous fungi. The literature reports hydrocarbon biodegradation as a common characteristic of Chaetothyrialean fungi, especially in the derived Herpotrichiellaceae family, which includes the *Cladophialophora* and *Exophiala* genera. Therefore, it was expected that a higher number of representatives of this group would be found as potential toluene degraders. All selected strains belonging to the Pleosporales order were obtained from water samples under the influence of an oil refinery, while *Cladosporium* sp. strains (Cladosporiales) were isolated from ant exoskeletons. It is quite unexpected that the members of the Chaetothyriales that were found among the potential toluene-growing strains (Table 3) were the species *Exophiala dermatitidis* and *Cladophialophora devriesii*, while strains originally related to hydrocarbon-associated environments such as *E. xenobiotica* and *C. immunda*, for example, did not show significant growth on toluene. Reliable screening methodologies for evaluating the microbial assimilation potential of volatile substrates such as toluene are scarce. Several drawbacks have been identified, such as the difficulties to deal with volatile substrates that might diffuse through a wide range of plastic materials and the oligotrophic nature of many microorganisms so that they can grow on agar impurities [102].

Blasi et al. [103] designed a high-throughput microtiter plate assay method for screening the ability of 163 fungal strains from a culture collection to grow on different pollutants, including toluene provided through the gas phase in an enclosed desiccator containing a beaker filled with this compound. The assimilation of toluene was inferred upon the analysis of cell growth by taking optical density measurements at different times, and 25 strains were selected as positive for toluene growth on this basis. However, toluene assimilation could only be verified for two of these strains, belonging to *C. immunda* and *E. mesophila*, using chromatographic measurements of toluene biodegradation and carbon dioxide production.

This study provides an additional inexpensive and straightforward method for assessing the fungal potential for toluene assimilation of a collection of melanized fungi isolated from different hydrocarbon-related environments. In addition, the effect of the toluene concentration was tested by mixing it with dibutyl phthalate as a way to mitigate the toxicity of this compound. However, this attenuation was not needed for many strains, which were able to grow on toluene under saturated air/water conditions. It is well known that melanized fungi can survive and grow in extreme environments. They can deal with water and nutrient scarcity, low pH, different kinds of radiation, and the presence of xenobiotics. This tolerance is associated with their conspicuous melanization. Melanins are complex molecules with uncommon physical and chemical properties that guarantee protection and adaptation to several stressful processes, including the presence of toxicants such as the BTEX.

In their review, Cordero et al. [23] presented several studies which confirm that microbial melanins can be very useful to reach a sustainable future, given that they can act on radioprotection, bioremediation of xenobiotics, and heavy-metal-polluted sites. The only sensitive issue regarding melanized microorganisms is the fact that melanin may be associated with microbial virulence. Species that are opportunistic and pathogenic to humans and other animals have been reported in this group. Therefore, it is necessary to consider all aspects of biosecurity, aiming to select the non-virulent strains.

A notorious result from this study is that there was considerable mycelial growth for several strains when no carbon source was offered (NC; Table S1). Dolatabadi et al. [104] reported this same condition for strains of *E. xenobiotica* and *Phialophora americana* isolated from oil-contaminated soil and tested for BTEX assimilation. Satow et al. [32] and Zhao et al. [20] reported the growth ability of melanized fungi under nutrient paucity conditions, highlighting the oligotrophic nature of these microorganisms that included oligocarbonotrophic traits. Since 1980, studies aiming at the characterization of the oligotroph nature presented by several fungal strains have been performed, and they indicate the ability of these microorganisms to remove the carbon necessary for their survival from inorganic sources, even from atmospheric CO_2_. According to Wainwright et al. [105], fungi can grow oligotrophically without apparent involvement of lysis and the use of preformed hyphae. Parkinson et al. [106] found hyphae of *Fusarium oxysporum* growing oligotrophically without undergoing lysis, remaining intact and filled with cytoplasm from the inoculation point to the tips of actively growing hyphae. Zhdanova et al. [107] isolated melanized fungi in nuclear reactors and their cooling water. Dadachova et al. [108], working with fungi isolated from Chernobyl nuclear reactors, reported that they seemed to use their melanin to convert gamma radiation into chemical energy for their development. Onofri et al. [109] discussed the survivability of these microorganisms in conditions analogous to the extraterrestrial environment.

Fungal degradation of xenobiotics such as toluene involves the presence of specific metabolic pathways that act on the aromatic ring cleavage and its conversion into assimilable compounds as sources of carbon and energy [19,31]. The study of melanized fungi as potential aromatic biodegraders has intensified in the last few decades because of their historical potential and the recurrent isolation of their representatives from environments related to the presence of hydrocarbons. Results obtained in this study highlight the biodiversity of melanized fungi present in hydrocarbon-associated environments and their potential capacity for the assimilative biodegradation of toluene. Further studies will be performed with selected strains in order to confirm their capacity to grow on toluene and assess their potential uses in environmental biotechnology applications.

## 4. Conclusions

The findings of this study illustrate the biodiversity of melanized fungi that survive in different natural and anthropogenic hydrocarbon-rich environments, and also their capacity to tolerate and even grow on toluene, supplemented as the sole carbon and energy source at relatively low and high concentrations. This information might be useful for designing new bioindication tools on the hydrocarbon exposure of natural and anthropized environments. The isolated strain populations appeared to be rather specific from the studied environments and ranged from the lowest biodiversity in the garage ground spilled with motor oil, where all 47 isolated strains belonged to *E. dermatitidis*, a well-known opportunistic pathogen, to the high biodiversity of the refinery-impacted river, in which 25 distinct species were observed out of 43 isolated strains. The results also confirmed the preference of the Chaetothyriales for hydrocarbonaceous environments, and *Exophiala* spp. were isolated from all the studied samples. This genus contains several species that have been investigated thoroughly for the biofiltration of air polluted with volatile organic compounds, but that have also been isolated from clinical cases. The high tolerance of black fungi, well above that of most bacteria, to harsh and fluctuating environmental conditions makes them ideal catalysts for several bioremediation applications, but might also predispose them towards virulence. Interestingly, the most promising results for the potential of toluene assimilation in terms of biomass yield were obtained with species from the Pleosporales, Cladosporiales, and Xylariales, which are generally less biohazardous than those from the Chaetothyriales. The capacity of the newly isolated fungi to metabolize toluene as the sole source of carbon and energy needs to be verified by more precise growth experiments, but it might well be so that the range of hydrocarbonoclastic fungal species with biotechnological potential needs to be revised in the future.

## Figures and Tables

**Table 1:** Melanized fungi isolation sources including their description, location and isolation methods applied.

Substrate of Isolation	Description	Locality (DMS * Coordinates)	Isolation Method
Contaminated soil	Soil from a garage shop	22°26′13″ S; 47°34′4″ W	Oi flotation
	Land farming soil from an oil refinery	22°43′34″ S; 47°8′7″ W	Oil flotation
Plant material	Bark fragments of *Eucalyptus tereticornis*	22°24′34.37″ S; 47°32′28.98″ W	Oil flotation
Water samples	Water samples from a river related to the activity of an oil refinery	22°44′23″ S; 47°07′40″ W	Standard serial dilution
Insects	Exoskeletons of gynes and drones of Attini ants	22°50′6″ S; 48°26′1″ W	Oil flotation/Agar walk

* DMS—Degrees, Minutes and Seconds.

**Table 2:** Alpha diversity indexes of melanized fungi based on the sequenced isolated strains.

Environmental Sample	Isolation Method ^a^	No. of Strains	Observed Richness	ACE ^b^	Chao1	Shannon	Simpson
*Eucalyptus tereticornis* bark	OF	9	3	7.07143	4	0.98643	0.37037
*Atta capiguara* drone	OF, AW	13	5	20.7037	11	1.50588	0.49704
*Atta laevigata* drone	AW	11	4	7.22469	4.5	1.49111	0.54545
Garage soil	OF	47	1	1	1	0	0
*Atta capiguara* gyne	OF, AW	7	2	3.11111	2	0.59167	0.2449
*Atta laevigata* gyne	AW	4	4	error	10	2	0.75
Landfarming soil	OF	7	4	5.78667	4.5	1.84237	0.69388
Water sample	PP	43	25	72.4482	47.6667	4.255	0.92699

^a^ OF: oil flotation; AW: agar walk; PP: pour plate. ^b^ Abundance-based Coverage Estimator.

**Table 3:** Selected strains from the desiccator test according to the growth ratios calculated using the biomass produced by the melanized fungi when glucose, toluene, or no carbon source was offered. The selection includes only strains that more than quintupled the biomass production compared to the endogenous growth (NC) when toluene was supplied as the only carbon source (TL and TH).

Data on the Strains			Biomass (mg)				Ratios		
ID	Species	Substrate of Isolation	GC	NC	TH	TL	GC/NC	TH/NC	TL/NC
A55	*Westerdykella dispersa*	Water samples	81.9	1.4	14.5	17.9	58.5	10.4	12.8
A234	*Pestalotiopsis* sp.	Water samples	38.8	0.3	4.4	4.1	129.3	14.7	13.7
N34	*Cladosporium* sp.	*Atta capiguara* drone	36.9	0.4	8.1	4.2	92.3	20.3	10.5
F21	Melanized filamentous fungus	*Atta capiguara* drone	82.6	1.5	14.8	10.3	55.1	9.9	6.9
D4	*Cladophialophora devriesii*	*Eucalyptus tereticornis* bark	86.4	1.4	17.0	6.6	61.7	12.1	4.7
D5	*Cladophialophora devriesii*	*Eucalyptus tereticornis* bark	81.7	1.6	4.4	18.1	51.1	2.8	11.3
D15	Chaetothyriales sp.	*Eucalyptus tereticornis* bark	90.7	1.8	18.9	2.4	50.4	10.5	1.3
D31	*Exophiala dermatitidis*	Soil from machine shop	76.8	1.8	17.5	4.3	42.7	9.7	2.4
D153	*Exophiala dermatitidis*	Soil from machine shop	87.2	2.7	16.9	18.1	32.3	6.3	6.7
D180	*Exophiala dermatitis*	Soil from machine shop	27.3	2.0	3.0	34.5	13.7	1.5	17.3
A126	*Pleosporales* sp.	Water samples	5.9	1.2	6.1	6.6	4.9	5.1	5.5
A304	Melanized filamentous fungus	Water samples	67.8	1.6	4.7	15.9	42.4	2.9	9.9
A393	*Pleosporales* sp.	Water samples	22.5	1.8	17.7	6.8	12.5	9.8	3.8
N21	*Cladosporium* sp.	*Atta capiguara* drone	25.7	3.0	19.0	18.3	8.6	6.3	6.1
N85	*Cladosporium* sp.	*Atta laevigata* drone	21.4	2.4	16.5	17.3	8.9	6.9	7.2
N92	*Cladosporium* sp.	*Atta capiguara* gyne	34.6	2.7	16.6	17.5	12.8	6.1	6.5
N101	*Cladosporium* sp.	*Atta laevigata* drone	49.7	2.2	17.9	17.1	22.6	8.1	7.8
F18	*Cladosporium* sp.	*Atta capiguara* drone	31.2	3.1	17.5	17.3	10.1	5.6	5.6
NR7	*Pleosporales* sp.	Water samples	81.3	1.5	16.6	4.8	54.2	11.1	3.2

GC = positive control (glucose); NC = negative control; TH = toluene atmosphere at high concentration; TL = toluene atmosphere at low concentration.

## Data Availability

Data are available in supplementary materials (Figures S1 and S2, Table S1).

## Supplementary Materials

The following are available online at https://www.mdpi.com/article/10.3390/microorganisms9051008/s1, Table S1. The following table contains values of biomass production in milligrams (mg) for the 200 strains submitted to the toluene assimilation test in each treatment, growth ratios, as well as their identification and source. GC = biomass produced with glucose as the carbon source; NC = biomass produced in the absence of a carbon source; TL = toluene as the sole carbon source added with dibutyl phthalate; TH = toluene as the sole carbon source. Figure S1. Phylogenetic analysis using sequences of strains from the Chaetothyriales order. Highlighted strains are representatives obtained in this study. Accession numbers are presented before taxon names for sequences collected at the Genbank. TYPE = Type strain material. The evolutionary history was inferred using the Maximum Likelihood method and the Kimura 2-parameter model (Kimura, 1980). The percentage of trees in which the associated taxa clustered together is shown next to the branches. Initial trees for the heuristic search were obtained automatically by applying the Neighbor-Joining and BioNJ algorithms to a matrix of pairwise distances estimated using the Maximum Composite Likelihood (MCL) approach, then selecting the topology with the highest log-likelihood value. The tree is drawn to scale, with branch lengths measured in the number of substitutions per site. This analysis involved 71 nucleotide sequences. There were a total of 643 positions in the final dataset. Figure S2. Phylogenetic analysis using sequences of strains from the orders Cladosporiales and Pleosporales. Highlighted strains are representatives obtained in this study. Accession numbers are presented before taxon names for sequences collected at the Genbank. TYPE = Type strain material. The evolutionary history was inferred using the Maximum Likelihood method and the Kimura 2-parameter model (Kimura, 1980). The percentage of trees in which the associated taxa clustered together is shown next to the branches. Initial trees for the heuristic search were obtained automatically by applying the Neighbor-Joining and BioNJ algorithms to a matrix of pairwise distances estimated using the Maximum Composite Likelihood (MCL) approach, then selecting the topology with the highest log-likelihood value. The tree is drawn to scale, with branch lengths measured in the number of substitutions per site. This analysis involved 39 nucleotide sequences. There were a total of 606 positions in the final dataset.
