# Supplementary material for: Black Fungi and Hydrocarbons: An Environmental Survey for Alkylbenzene Assimilation
Source: Microorganisms. 2021 May 7;9(5):1008. doi: 10.3390/microorganisms9051008 (PMC8151820; doi:10.3390/microorganisms9051008)
Supplement: Supplementary file 1 [file microorganisms-09-01008-s001.zip › Table S1.pdf]

Table S1. The following table contains values of biomass production in milligrams (mg) for the 200 strains submitted to the toluene assimilation test in each treatment, growth ratios, as well as their identification and source. GC = biomass produced with glucose as the carbon source; NC = biomass produced in the absence of a carbon source; TL = toluene as the sole carbon source added with dibutyl phthalate; TH = toluene as the sole carbon source.

Sample ID	Identification	Substrate	Isol. Method	Characterization	GC	NC	TL	TH	GC/NC	TH/NC	TL/NC
D4	*Cladophialophora devriesii*	*Eucalyptus tereticornis* bark	Oil flotation	Molecular	86.4	1.4	6.6	17.0	61.7	12.1	4.7
D5	*Cladophialophora devriesii*	*Eucalyptus tereticornis* bark	Oil flotation	Molecular	81.7	1.6	18.1	4.4	51.1	2.8	11.3
D15	Chaetothyriales species	*Eucalyptus tereticornis* bark	Oil flotation	Morphology	90.7	1.8	2.4	18.9	50.4	10.5	1.3
D16	*Cladophialophora devriesii*	*Eucalyptus tereticornis* bark	Oil flotation	Molecular	35.4	18.6	17.3	6.1	1.9	0.3	0.9
D23	*Exophiala dermatitis*	Soil from garage shop	Oil flotation	Molecular	52.2	10.3	5.1	16.7	5.1	1.6	0.5
D25	*Exophiala dermatitis*	Soil from garage shop	Oil flotation	Molecular	12.8	2.0	2.6	17.8	6.4	8.9	1.3
D29	*Exophiala dermatitis*	Soil from garage shop	Oil flotation	Molecular	35.8	5.0	8.0	18.2	7.2	3.6	1.6
D30	*Exophiala dermatitis*	Soil from garage shop	Oil flotation	Molecular	8.2	15.2	4.3	19.3	0.5	1.3	0.3
D31	*Exophiala dermatitis*	Soil from garage shop	Oil flotation	Molecular	76.8	1.8	4.3	17.5	42.7	9.7	2.4
D41	Chaetothyriales species	Soil from garage shop	Oil flotation	Morphology	84.5	10.0	3.2	5.3	8.5	0.5	0.3
D48	Chaetothyriales species	Soil from garage shop	Oil flotation	Morphology	87.1	16.8	3.9	4.1	5.2	0.2	0.2
D51	*Exophiala dermatitis*	Soil from garage shop	Oil flotation	Molecular	108.3	3.1	5.4	16.3	34.9	5.3	1.7
D53	*Exophiala dermatitis*	Soil from garage shop	Oil flotation	Molecular	76.7	2.8	11.9	8.6	27.4	3.1	4.3
D59	*Exophiala dermatitis*	Soil from garage shop	Oil flotation	Molecular	58.5	3.6	4.4	5.0	16.3	1.4	1.2
D63	*Exophiala dermatitis*	Soil from garage shop	Oil flotation	Molecular	48.0	3.4	15.0	17.8	14.1	5.2	4.4
D65	*Cladophialophora devriesii*	*Eucalyptus tereticornis* bark	Oil flotation	Molecular	84.7	6.1	6.2	6.4	13.9	1.0	1.0
D73	*Cladophialophora devriesii*	*Eucalyptus tereticornis* bark	Oil flotation	Molecular	80.8	2.9	1.6	9.9	27.9	3.4	0.6
D74	*Cladophialophora devriesii*	*Eucalyptus tereticornis* bark	Oil flotation	Molecular	86.0	19.7	2.8	6.1	4.4	0.3	0.1
D84	Chaetothyriales species	*Eucalyptus tereticornis* bark	Oil flotation	Morphology	91.7	3.9	4.3	6.3	23.5	1.6	1.1
D90	*Cladosporium* sp.	*Eucalyptus tereticornis* bark	Oil flotation	Molecular	88.2	18.2	2.8	10.6	4.8	0.6	0.2
D91	*Cladophialophora devriesii*	*Eucalyptus tereticornis* bark	Oil flotation	Molecular	41.0	3.4	4.6	6.8	12.1	2.0	1.4
D103	*Cladophialophora devriesii*	*Eucalyptus tereticornis* bark	Oil flotation	Molecular	86.8	18.6	6.2	11.4	4.7	0.6	0.3
D111	*Rhinocladiella atrovirens*	*Eucalyptus tereticornis* bark	Oil flotation	Molecular	81.3	4.1	7.5	16.5	19.8	4.0	1.8
D112	Chaetothyriales species	*Eucalyptus tereticornis* bark	Oil flotation	Morphology	78.2	8.3	3.1	16.7	9.4	2.0	0.4
D115	*Exophiala dermatitis*	Soil from garage shop	Oil flotation	Molecular	33.7	6.6	6.6	17.2	5.1	2.6	1.0
D116	*Exophiala dermatitis*	Soil from garage shop	Oil flotation	Molecular	83.0	10.6	3.6	16.8	7.8	1.6	0.3
D119	*Exophiala dermatitis*	Soil from garage shop	Oil flotation	Molecular	37.4	2.9	8.1	16.8	12.9	5.8	2.8
D121	Chaetothyriales species	Soil from garage shop	Oil flotation	Morphology	18.0	4.0	2.5	6.9	4.5	1.7	0.6
D122	*Exophiala dermatitis*	Soil from garage shop	Oil flotation	Molecular	85.8	9.8	4.5	6.3	8.8	0.6	0.5
D123	*Exophiala dermatitis*	Soil from garage shop	Oil flotation	Molecular	39.8	3.3	4.6	3.5	12.1	1.1	1.4
D127	*Exophiala dermatitis*	Soil from garage shop	Oil flotation	Molecular	78.3	2.7	4.0	4.9	29.0	1.8	1.5
D128	*Exophiala dermatitis*	Soil from garage shop	Oil flotation	Molecular	30.6	3.8	5.8	2.5	8.1	0.7	1.5

D129	*Exophiala dermatitis*	Soil from garage shop	Oil flotation	Molecular	86.8	3.1	4.5	5.9	28.0	1.9	1.5
D131	*Exophiala dermatitis*	Soil from garage shop	Oil flotation	Molecular	83.6	2.9	5.1	4.5	28.8	1.6	1.8
D132	*Exophiala dermatitis*	Soil from garage shop	Oil flotation	Molecular	21.1	3.9	2.1	3.3	5.4	0.8	0.5
D133	*Exophiala dermatitis*	Soil from garage shop	Oil flotation	Molecular	84.1	4.4	5.0	16.5	19.1	3.8	1.1
D135	*Exophiala dermatitis*	Soil from garage shop	Oil flotation	Molecular	85.0	3.0	17.9	4.8	28.3	1.6	6.0
D137	*Exophiala dermatitis*	Soil from garage shop	Oil flotation	Molecular	11.9	17.8	3.2	9.7	0.7	0.5	0.2
D138	*Exophiala dermatitis*	Soil from garage shop	Oil flotation	Molecular	83.9	3.0	3.5	7.5	28.0	2.5	1.2
D139	*Exophiala dermatitis*	Soil from garage shop	Oil flotation	Molecular	70.3	5.2	7.3	15.5	13.5	3.0	1.4
D141	*Exophiala dermatitis*	Soil from garage shop	Oil flotation	Molecular	20.6	12.5	6.1	5.1	1.6	0.4	0.5
D146	*Exophiala dermatitis*	Soil from garage shop	Oil flotation	Molecular	32.2	2.5	5.8	18.1	12.9	7.2	2.3
D149	*Exophiala dermatitis*	Soil from garage shop	Oil flotation	Molecular	22.2	4.5	3.1	9.9	4.9	2.2	0.7
D151	*Exophiala dermatitis*	Soil from garage shop	Oil flotation	Molecular	94.1	17.9	3.9	17.8	5.3	1.0	0.2
D153	*Exophiala dermatitis*	Soil from garage shop	Oil flotation	Molecular	87.2	2.7	18.1	16.9	32.3	6.3	6.7
D154	*Exophiala dermatitis*	Soil from garage shop	Oil flotation	Molecular	34.7	2.7	3.2	6.0	12.9	2.2	1.2
D157	*Exophiala dermatitis*	Soil from garage shop	Oil flotation	Molecular	19.0	3.7	18.1	15.0	5.1	4.1	4.9
D158	*Exophiala dermatitis*	Soil from garage shop	Oil flotation	Molecular	80.0	2.3	10.7	7.7	34.8	3.3	4.7
D160	*Exophiala dermatitis*	Soil from garage shop	Oil flotation	Molecular	93.3	16.5	17.8	16.6	5.7	1.0	1.1
D162	*Exophiala dermatitis*	Soil from garage shop	Oil flotation	Molecular	74.7	7.6	4.4	17.0	9.8	2.2	0.6
D164	*Exophiala dermatitis*	Soil from garage shop	Oil flotation	Molecular	17.0	2.4	8.4	17.5	7.1	7.3	3.5
D165	*Exophiala dermatitis*	Soil from garage shop	Oil flotation	Molecular	11.6	3.8	6.2	17.3	3.1	4.6	1.6
D168	*Exophiala dermatitis*	Soil from garage shop	Oil flotation	Molecular	85.6	1.9	4.9	4.9	45.1	2.6	2.6
D169	*Exophiala dermatitis*	Soil from garage shop	Oil flotation	Molecular	9.3	4.7	5.4	16.3	2.0	3.5	1.1
D170	Chaetothyriales species	Soil from garage shop	Oil flotation	Molecular	93.2	3.2	6.3	21.5	29.1	6.7	2.0
D171	*Exophiala dermatitis*	Soil from garage shop	Oil flotation	Molecular	86.9	4.5	15.3	12.7	19.3	2.8	3.4
D173	Chaetothyriales species	Soil from garage shop	Oil flotation	Morphology	11.7	1.7	3.3	14.1	6.9	8.3	1.9
D180	*Exophiala dermatitis*	Soil from garage shop	Oil flotation	Molecular	27.3	2.0	34.5	3.0	13.7	1.5	17.3
D183	*Exophiala dermatitis*	Soil from garage shop	Oil flotation	Molecular	30.5	2.8	7.0	7.2	10.9	2.6	2.5
D192	*Exophiala dermatitis*	Soil from garage shop	Oil flotation	Molecular	19.9	17.6	8.3	8.0	1.1	0.5	0.5
D199	*Exophiala dermatitis*	Soil from garage shop	Oil flotation	Molecular	85.9	7.2	11.5	17.3	11.9	2.4	1.6
D200	*Exophiala dermatitis*	Soil from garage shop	Oil flotation	Molecular	9.2	3.5	17.4	5.6	2.6	1.6	5.0
D201	*Exophiala dermatitis*	Soil from garage shop	Oil flotation	Molecular	129.4	1.4	3.3	9.2	92.4	6.6	2.4
D203	*Exophiala dermatitis*	Soil from garage shop	Oil flotation	Molecular	39.3	2.5	17.2	11.2	15.7	4.5	6.9
D207	*Exophiala dermatitis*	Soil from garage shop	Oil flotation	Molecular	76.2	12.7	18.2	12.9	6.0	1.0	1.4
A6	*Cladosporium* sp.	Water samples	Pour plate	Morphology	41.6	16.7	5.8	18.1	2.5	1.1	0.3
A18	*Curvularia geniculata*	Water samples	Pour plate	Molecular	50.3	16.6	16.7	18.8	3.0	1.1	1.0

A28	*Cladosporium* sp.	Water samples	Pour plate	Morphology	41.7	15.3	5.7	9.3	2.7	0.6	0.4
A30b	*Phoma*-like	Water samples	Pour plate	Morphology	28.2	15.5	16.4	7.6	1.8	0.5	1.1
A48	*Westerdykella capitulum*	Water samples	Pour plate	Molecular	89.6	3.4	5.6	2.3	26.4	0.7	1.6
A54	Pleosporales species	Water samples	Pour plate	Morphology	62.2	2.8	3.1	8.6	22.2	3.1	1.1
A55	*Westerdykella dispersa*	Water samples	Pour plate	Molecular	81.9	1.4	17.9	14.5	58.5	10.4	12.8
A58	Pleosporales species	Water samples	Pour plate	Morphology	51.0	4.4	17.9	2.9	11.6	0.7	4.1
A64	*Phoma*-like	Water samples	Pour plate	Molecular	85.6	15.7	10.1	7.7	5.5	0.5	0.6
A65	Pleosporales species	Water samples	Pour plate	Morphology	26.1	16.7	3.1	16.8	1.6	1.0	0.2
A67	*Microsphaeropsis arundinis*	Water samples	Pour plate	Molecular	26.4	2.7	5.9	6.4	9.8	2.4	2.2
A70	*Phoma*-like	Water samples	Pour plate	Morphology	29.8	16.6	17.7	19.3	1.8	1.2	1.1
A77	*Westerdykella dispersa*	Water samples	Pour plate	Molecular	87.6	15.7	6.5	5.3	5.6	0.3	0.4
A78	*Microsphaeropsis arundinis*	Water samples	Pour plate	Molecular	52.2	4.0	2.9	5.6	13.1	1.4	0.7
A80	Pleosporales species	Water samples	Pour plate	Morphology	66.4	16.5	6.9	4.1	4.0	0.2	0.4
A94	*Microsphaeropsis arundinis*	Water samples	Pour plate	Molecular	63.5	3.3	16.9	5.2	19.2	1.6	5.1
A95	Pleosporales species	Water samples	Pour plate	Morphology	22.9	3.2	7.4	7.3	7.2	2.3	2.3
A107	Melanized filamentous fungi	Water samples	Pour plate	Morphology	29.2	4.5	17.3	4.8	6.5	1.1	3.8
A125	*Microsphaeropsis arundinis*	Water samples	Pour plate	Molecular	35.3	3.4	3.8	6.6	10.4	1.9	1.1
A126	Pleosporales species	Water samples	Pour plate	Morphology	5.9	1.2	6.6	6.1	4.9	5.1	5.5
A130	*Cochliobolus kusanoi*	Water samples	Pour plate	Molecular	18.6	3.9	4.6	17.6	4.8	4.5	1.2
A135	Melanized filamentous fungi	Water samples	Pour plate	Morphology	98.4	15.3	10.5	5.3	6.4	0.3	0.7
A144	*Paraphaeosphaeria* sp.	Water samples	Pour plate	Molecular	39.2	16.7	18.3	4.4	2.3	0.3	1.1
A149	*Cladosporium* sp.	Water samples	Pour plate	Molecular	31.6	15.8	18.5	6.8	2.0	0.4	1.2
A151	*Microsphaeropsis arundinis*	Water samples	Pour plate	Molecular	64.2	3.6	5.8	19.0	17.8	5.3	1.6
A165	*Westerdykella capitulum*	Water samples	Pour plate	Molecular	92.5	2.7	11.1	4.5	34.3	1.7	4.1
A172	*Paraphaeosphaeria* sp.	Water samples	Pour plate	Molecular	67.6	1.1	5.5	2.9	61.5	2.6	5.0
A177	Pleosporales species	Water samples	Pour plate	Morphology	87.6	10.5	6.7	9.1	8.3	0.9	0.6
A179	Pleosporales species	Water samples	Pour plate	Molecular	29.7	6.3	3.7	9.3	4.7	1.5	0.6
A184	*Didymella* sp.	Water samples	Pour plate	Molecular	85.2	18.2	12.1	14.6	4.7	0.8	0.7
A189	*Cladosporium* sp.	Water samples	Pour plate	Morphology	41.0	6.4	8.9	17.1	6.4	2.7	1.4
A234	*Pestalotiopsis* sp.	Water samples	Pour plate	Molecular	38.8	0.3	4.1	4.4	129.3	14.7	13.7
A240	Pleosporales species	Water samples	Pour plate	Morphology	27.1	16.3	4.1	5.2	1.7	0.3	0.3
A237	*Westerdykella capitulum*	Water samples	Pour plate	Molecular	43.9	16.3	8.4	6.3	2.7	0.4	0.5
A246	*Cladosporium* sp.	Water samples	Pour plate	Molecular	58.1	15.9	11.1	17.6	3.7	1.1	0.7
A259	*Cladosporium* sp.	Water samples	Pour plate	Morphology	48.2	16.9	18.1	17.3	2.9	1.0	1.1
A272	*Cladosporium* sp.	Water samples	Pour plate	Molecular	47.3	7.0	16.5	16.3	6.8	2.3	2.4

A275	Pleosporales species	Water samples	Pour plate	Molecular	28.5	16.5	19.0	3.9	1.7	0.2	1.2
A304	Melanized filamentous fungi	Water samples	Pour plate	Morphology	67.8	1.6	15.9	4.7	42.4	2.9	9.9
A305	Melanized filamentous fungi	Water samples	Pour plate	Morphology	38.6	16.3	5.8	17.4	2.4	1.1	0.4
A307	*Rhinocladiella similis*	Water samples	Pour plate	Molecular	78.9	16.3	17.1	17.4	4.8	1.1	1.0
A316	*Didymella* sp.	Water samples	Pour plate	Molecular	27.3	17.6	5.9	13.7	1.6	0.8	0.3
A327	Pleosporales species	Water samples	Pour plate	Morphology	80.7	6.8	6.3	3.8	11.9	0.6	0.9
A335	Pleosporales species	Water samples	Pour plate	Morphology	42.8	14.3	15.5	10.9	3.0	0.8	1.1
A340	*Pseudallescheria boydii*	Water samples	Pour plate	Molecular	19.3	16.4	9.5	17.4	1.2	1.1	0.6
A348	*Exophiala spinifera*	Water samples	Pour plate	Molecular	44.2	3.8	13.0	7.5	11.6	2.0	3.4
A356	*Epicoccum* sp.	Water samples	Pour plate	Molecular	30.7	17.2	11.1	22.6	1.8	1.3	0.6
A373	Pleosporales species	Water samples	Pour plate	Molecular	31.3	1.8	12.0	4.0	17.4	2.2	6.7
A374	Pleosporales species	Water samples	Pour plate	Molecular	74.5	2.6	8.1	4.9	28.7	1.9	3.1
A390	Pleosporales species	Water samples	Pour plate	Morphology	31.0	3.4	1.5	8.6	9.1	2.5	0.4
A393	Pleosporales species	Water samples	Pour plate	Morphology	22.5	1.8	6.8	17.7	12.5	9.8	3.8
A398	*Microsphaeropsis arundinis*	Water samples	Pour plate	Molecular	114.5	18.0	5.0	19.3	6.4	1.1	0.3
A418	Pleosporales species	Water samples	Pour plate	Molecular	40.5	2.1	2.5	5.8	19.3	2.8	1.2
A420	Pleosporales species	Water samples	Pour plate	Morphology	40.4	2.4	8.1	3.6	16.8	1.5	3.4
A424	*Westerdykella capitulum*	Water samples	Pour plate	Molecular	112.1	15.6	5.6	18.0	7.2	1.2	0.4
A457	Pleosporales species	Water samples	Pour plate	Morphology	23.7	9.3	4.8	14.2	2.5	1.5	0.5
N18	*Cladosporium* sp.	*Atta capiguara* gyne	Agar walk	Molecular	40.2	20.4	15.8	9.0	2.0	0.4	0.8
N19	*Cladosporium* sp.	*Atta capiguara* gyne	Agar walk	Molecular	35.7	17.3	2.9	19.2	2.1	1.1	0.2
N21	*Cladosporium* sp.	*Atta capiguara* drone	Oil flotation	Morphology	25.7	3.0	18.3	19.0	8.6	6.3	6.1
N24	*Cladosporium* sp.	*Atta capiguara* drone	Oil flotation	Morphology	34.1	3.2	3.1	5.6	10.7	1.8	1.0
N26	*Cladosporium* sp.	*Atta capiguara* gyne	Oil flotation	Morphology	36.2	4.7	3.6	5.8	7.7	1.2	0.8
N27	*Cladosporium* sp.	*Atta capiguara* drone	Agar walk	Morphology	35.6	4.3	5.2	18.6	8.3	4.3	1.2
N32	*Cladosporium* sp.	*Atta capiguara* drone	Agar walk	Molecular	50.4	3.4	4.0	0.4	14.8	0.1	1.2
N34	*Cladosporium* sp.	*Atta capiguara* drone	Agar walk	Morphology	36.9	0.4	4.2	8.1	92.3	20.3	10.5
N36	*Cladosporium* sp.	*Atta capiguara* drone	Agar walk	Morphology	32.0	14.9	12.8	16.1	2.1	1.1	0.9
N44	*Cladosporium* sp.	*Atta capiguara* drone	Agar walk	Morphology	38.0	3.5	4.8	13.1	10.9	3.7	1.4
N45	*Cochliobolus geniculatus*	*Atta capiguara* drone	Agar walk	Molecular	52.3	3.8	5.7	14.7	13.8	3.9	1.5
N47	*Cladosporium* sp.	*Atta capiguara* drone	Agar walk	Molecular	45.2	15.6	4.0	5.6	2.9	0.4	0.3
N49	Pleosporales species	*Atta laevigata* drone	Agar walk	Morphology	81.0	1.8	4.9	8.7	45.0	4.8	2.7
N53	*Cladophialophora mycetomatis*	*Atta laevigata* drone	Agar walk	Molecular	37.3	7.5	3.9	8.8	5.0	1.2	0.5
N58	*Verruconis verruculosum*	*Atta laevigata* drone	Agar walk	Molecular	84.4	5.8	3.5	4.5	14.6	0.8	0.6
N61	*Cochliobolus geniculatus*	*Atta laevigata* gyne	Agar walk	Molecular	26.9	16.6	3.8	7.6	1.6	0.5	0.2

N64	*Exophiala bergeri*	*Atta laevigata* gyne	Agar walk	Molecular	70.0	6.3	3.5	1.7	11.1	0.3	0.6
N66	*Cladosporium* sp.	*Atta laevigata* drone	Agar walk	Morphology	51.1	5.6	15.3	6.1	9.1	1.1	2.7
N67	*Xenopenidiella formica*	*Atta laevigata* drone	Agar walk	Molecular	64.9	4.3	17.7	15.0	15.1	3.5	4.1
N72	Pleosporales species	*Atta capiguara* gyne	Agar walk	Morphology	26.4	3.1	4.5	0.4	8.5	0.1	1.5
N73	*Cladosporium* sp.	*Atta capiguara* gyne	Agar walk	Morphology	34.1	7.9	4.0	7.7	4.3	1.0	0.5
N74	*Cladosporium* sp.	*Atta laevigata* gyne	Agar walk	Molecular	39.8	8.9	4.4	17.8	4.5	2.0	0.5
N75	*Cladosporium* sp.	*Atta capiguara* drone	Agar walk	Molecular	35.1	17.0	3.7	18.4	2.1	1.1	0.2
N76	*Cladosporium* sp.	*Atta capiguara* drone	Agar walk	Molecular	36.9	2.2	5.6	2.8	16.8	1.3	2.5
N77	*Cladosporium* sp.	*Atta capiguara* drone	Agar walk	Molecular	36.9	11.9	17.1	0.8	3.1	0.1	1.4
N79	*Cladosporium* sp.	*Atta capiguara* drone	Oil flotation	Morphology	36.9	5.2	17.4	17.2	7.1	3.3	3.3
N80	*Cladosporium* sp.	*Atta laevigata* drone	Agar walk	Molecular	40.3	18.4	17.4	17.4	2.2	0.9	0.9
N82	*Cladosporium* sp.	*Atta laevigata* drone	Agar walk	Molecular	31.2	17.6	45.8	16.9	1.8	1.0	2.6
N83	*Cladosporium* sp.	*Atta capiguara* gyne	Oil flotation	Morphology	42.3	2.6	4.3	6.4	16.3	2.5	1.7
N84	*Cladosporium* sp.	*Atta laevigata* drone	Agar walk	Molecular	51.8	3.5	4.2	4.5	14.8	1.3	1.2
N85	*Cladosporium* sp.	*Atta laevigata* drone	Agar walk	Molecular	21.4	2.4	17.3	16.5	8.9	6.9	7.2
N86	*Cladosporium* sp.	*Atta laevigata* drone	Agar walk	Molecular	70.0	3.4	5.9	4.8	20.6	1.4	1.7
N87	*Xenopenidiella inflata*	*Atta laevigata* drone	Agar walk	Molecular	75.1	17.9	4.4	16.0	4.2	0.9	0.2
N89	*Xenopenidiella laevigata*	*Atta laevigata* gyne	Agar walk	Molecular	32.6	11.0	3.6	16.7	3.0	1.5	0.3
N92	*Cladosporium* sp.	*Atta capiguara* gyne	Agar walk	Morphology	34.6	2.7	17.5	16.6	12.8	6.1	6.5
N101	*Cladosporium* sp.	*Atta laevigata* drone	Agar walk	Molecular	49.7	2.2	17.1	17.9	22.6	8.1	7.8
N110	*Cladosporium* sp.	*Atta laevigata* drone	Agar walk	Molecular	57.8	16.9	16.8	8.4	3.4	0.5	1.0
F3	*Cladosporium* sp.	*Atta capiguara* drone	Oil flotation	Molecular	44.6	14.9	16.6	15.5	3.0	1.0	1.1
4F1	Capnodiales species	*Atta capiguara* drone	Oil flotation	Morphology	90.3	2.4	18.9	2.5	37.6	1.0	7.9
4F2	Capnodiales species	*Atta capiguara* drone	Oil flotation	Morphology	85.9	2.3	4.8	6.9	37.3	3.0	2.1
F7	*Cladosporium* sp.	*Atta capiguara* gyne	Oil flotation	Molecular	38.8	24.2	17.6	17.7	1.6	0.7	0.7
F10	*Cladophialophora chaetospira*	*Atta capiguara* drone	Oil flotation	Molecular	81.3	14.9	8.4	16.1	5.5	1.1	0.6
F11	*Cladosporium* sp.	*Atta capiguara* drone	Oil flotation	Molecular	36.0	2.6	7.9	16.3	13.8	6.3	3.0
F12	*Cladosporium* sp.	*Atta capiguara* drone	Oil flotation	Molecular	38.1	18.3	17.9	15.8	2.1	0.9	1.0
F14	*Cladosporium* sp.	*Atta capiguara* gyne	Oil flotation	Molecular	534.9	2.2	18.0	4.4	243.1	2.0	8.2
F18	*Cladosporium* sp.	*Atta capiguara* drone	Oil flotation	Molecular	31.2	3.1	17.3	17.5	10.1	5.6	5.6
F21	Melanized filamentous fungi	*Atta capiguara* drone	Oil flotation	Morphology	82.6	1.5	10.3	14.8	55.1	9.9	6.9
F22	Melanized filamentous fungi	*Atta capiguara* gyne	Oil flotation	Morphology	79.1	18.4	15.5	5.1	4.3	0.3	0.8
F25	*Pithomyces*-like	*Atta capiguara* drone	Oil flotation	Morphology	77.6	5.8	3.5	5.9	13.4	1.0	0.6
F26	*Exophiala spinifera*	*Atta capiguara* drone	Oil flotation	Molecular	102.2	4.1	5.6	17.0	24.9	4.1	1.4
F27	Melanized filamentous fungi	*Atta capiguara* gyne	Oil flotation	Morphology	87.3	16.8	2.0	7.6	5.2	0.5	0.1

F29	*Cladosporium* sp.	*Atta capiguara* gyne	Oil flotation	Molecular	34.1	17.3	17.7	3.4	2.0	0.2	1.0
F30	Chaetothyriales species	*Atta capiguara* gyne	Oil flotation	Morphology	31.8	12.1	19.3	16.5	2.6	1.4	1.6
F33	*Cladosporium* sp.	*Atta capiguara* drone	Oil flotation	Molecular	31.2	7.9	17.8	16.0	3.9	2.0	2.3
F34	*Cladosporium* sp.	*Atta capiguara* gyne	Oil flotation	Molecular	34.0	4.1	3.3	16.3	8.3	4.0	0.8
F35	*Cladosporium* sp.	*Atta capiguara* gyne	Oil flotation	Molecular	59.8	18.3	5.5	16.0	3.3	0.9	0.3
NR1	*Exophiala spinifera*	Water samples	Pour plate	Molecular	74.6	4.7	2.1	4.6	15.9	1.0	0.4
NR2	*Exophiala attenuata*	Water samples	Pour plate	Molecular	91.1	4.8	6.0	7.9	19.0	1.6	1.3
NR3	*Exophiala heteromorpha*	Water samples	Pour plate	Molecular	65.8	2.4	5.2	5.1	27.4	2.1	2.2
NR4	*Exophiala alcalophila*	Water samples	Pour plate	Molecular	92.9	5.0	3.1	17.7	18.6	3.5	0.6
NR7	Pleosporales species	Water samples	Pour plate	Molecular	81.3	1.5	4.8	16.6	54.2	11.1	3.2
NR8	*Exophiala attenuata*	Water samples	Pour plate	Molecular	25.2	13.2	3.6	16.3	1.9	1.2	0.3
NR10	Pleosporales species	Water samples	Pour plate	Molecular	22.2	15.4	10.7	16.8	1.4	1.1	0.7
NR11	*Didymella glomerata*	Water samples	Pour plate	Molecular	67.1	4.2	4.8	16.1	16.0	3.8	1.1
NR12	Pleosporales species	Water samples	Pour plate	Molecular	34.7	6.6	17.3	6.5	5.3	1.0	2.6
NR16	Pleosporales species	Water samples	Pour plate	Molecular	29.0	0.0	6.0	11.1	0.0	0.0	0.0
NR35	*Exophiala spinifera*	Water samples	Pour plate	Molecular	32.6	1.4	12.2	4.9	23.3	3.5	8.7
NR36	*Curvularia platzii*	Water samples	Pour plate	Molecular	25.6	16.8	17.2	17.2	1.5	1.0	1.0
M4	*Cladophialophora minourae*	Landfarming soil	Oil flotation	Molecular	91.4	16.8	2.2	3.1	5.4	0.2	0.1
M9	Chaetothyriales species	Landfarming soil	Oil flotation	Morphology	49.2	10.2	10.8	11.3	4.8	1.1	1.1
M14	*Cladophialophora immunda*	Landfarming soil	Oil flotation	Molecular	43.4	10.4	2.5	10.4	4.2	1.0	0.2
M46-1	*Cladophialophora immunda*	Landfarming soil	Oil flotation	Molecular	48.1	20.2	7.0	9.7	2.4	0.5	0.3
M68	Chaetothyriales species	Landfarming soil	Oil flotation	Morphology	31.6	10.6	4.3	6.2	3.0	0.6	0.4
M69	*Exophiala xenobiotica*	Landfarming soil	Oil flotation	Molecular	47.6	9.8	4.2	10.2	4.9	1.0	0.4
M75	*Cladosporium* sp.	Landfarming soil	Oil flotation	Molecular	32.4	10.3	11.3	2.1	3.1	0.2	1.1
M87	*Cladophialophora* sp.	Landfarming soil	Oil flotation	Morphology	46.3	9.8	8.3	7.1	4.7	0.7	0.8
M114	*Cladophialophora immunda*	Landfarming soil	Oil flotation	Molecular	44.7	9.8	11.3	10.8	4.6	1.1	1.2
M109	*Cladophialophora immunda*	Landfarming soil	Oil flotation	Molecular	55.3	11.3	3.3	9.3	4.9	0.8	0.3
M116	*Cladosporium* sp.	Landfarming soil	Oil flotation	Morphology	29.9	10.6	11.3	9.9	2.8	0.9	1.1
