# Supplementary figures and images for: Black Fungi and Hydrocarbons: An Environmental Survey for Alkylbenzene Assimilation
Source: Microorganisms. 2021 May 7;9(5):1008. doi: 10.3390/microorganisms9051008 (PMC8151820; doi:10.3390/microorganisms9051008)

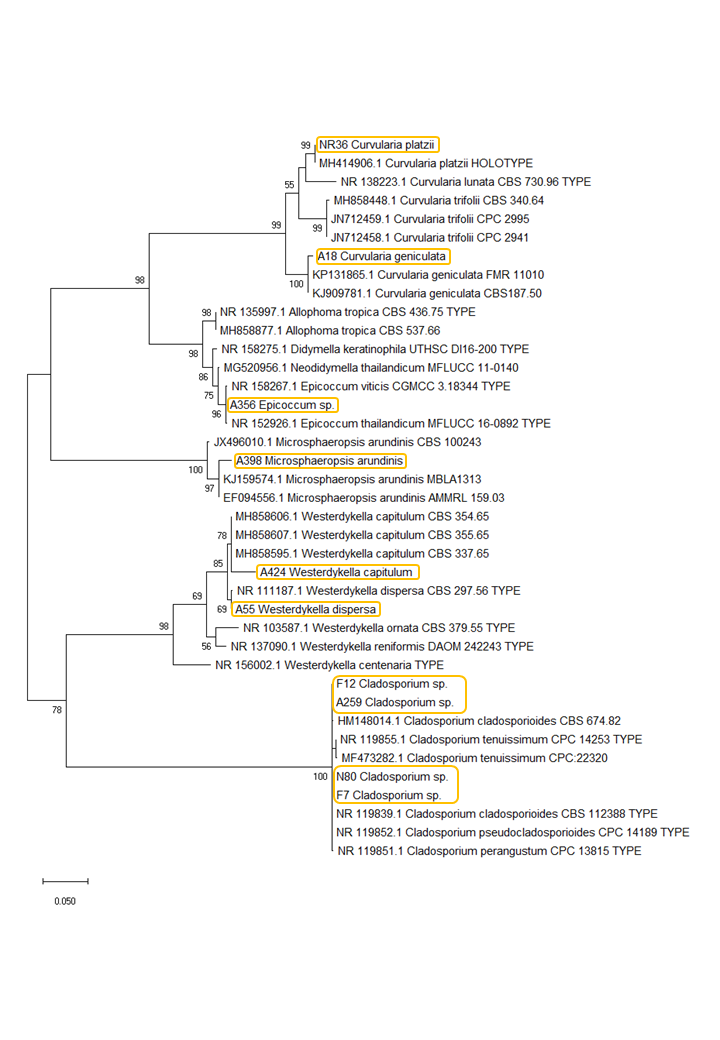

Supplement: Supplementary file 1 [file microorganisms-09-01008-s001.zip › Supl_Material_Fig_S2.png]

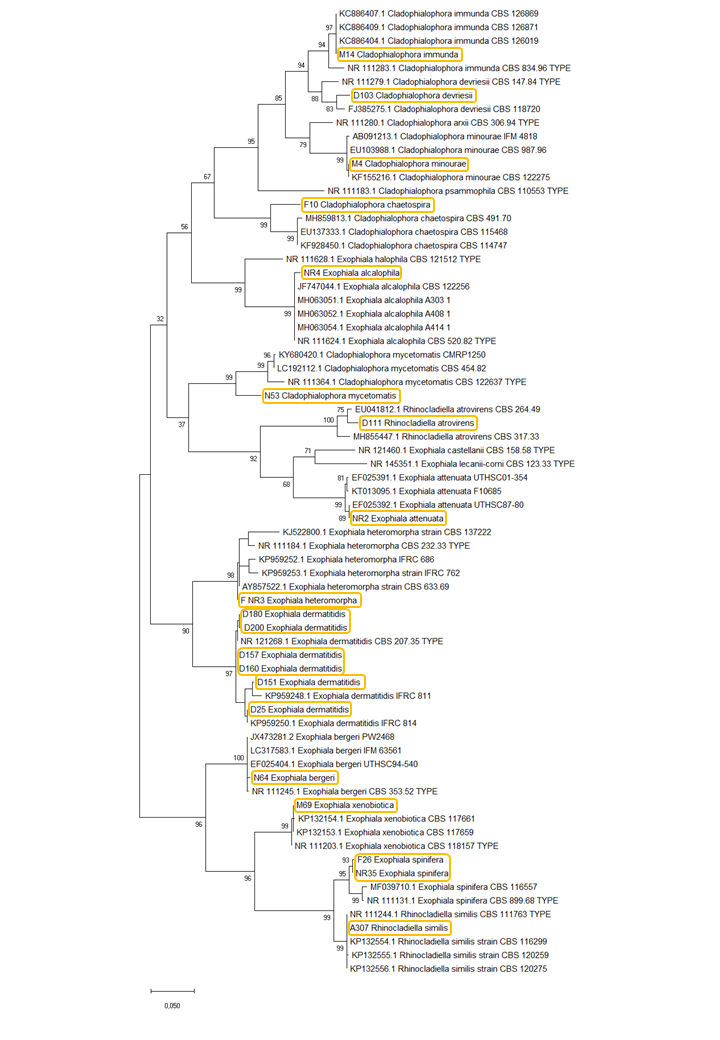

Supplement: Supplementary file 1 [file microorganisms-09-01008-s001.zip › Supl_Material_Fig_S1.png]
